# Heart–Brain Temporal Coupling as a Candidate Biomarker of Self-Congruency

**DOI:** 10.3390/biomedicines14030548

**Published:** 2026-02-27

**Authors:** Nicolas Bourdillon, Sébastien Urben, Nina Rimorini, Alicia Rey, Cyril Besson, Jean-Baptiste Ledoux, Yasser Alemán-Gómez, Eleonora Fornari, Solange Denervaud

**Affiliations:** 1 Institute of Sport Sciences, University of Lausanne (UNIL), 1015 Lausanne, Switzerland; nicolas.bourdillon@unil.ch (N.B.);; 2 Teaching and Research Unit in Physical Education and Sport (UER EPS), University of Teacher Education (HEP), 1007 Lausanne, Switzerland; 3 Division of Child and Adolescent Psychiatry (SUPEA), Department of Psychiatry, Lausanne University Hospital (CHUV-UNIL), 1011 Lausanne, Switzerland; 4 CIBM Center for Biomedical Imaging, Department of Radiology, Lausanne University Hospital (CHUV) and University of Lausanne (UNIL), 1015 Lausanne, Switzerland; nina.rimorini@chuv.ch (N.R.);; 5 Sports Medicine Center, Swiss Olympic Medical Center, Lausanne University Hospital (CHUV), 1011 Lausanne, Switzerland; 6 Department of Diagnostic and Interventional Radiology, Lausanne University Hospital (CHUV) and University of Lausanne (UNIL), 1011 Lausanne, Switzerland; 7 MRI imaging and technology, Polytechnical School of Lausanne, Swiss Federal Institute of Technology Lausanne (EPFL), 1015 Lausanne, Switzerland

**Keywords:** heart rate variability, neural dynamics variability, self-congruency, neurovisceral integration model, emotion regulation, mental health

## Abstract

**Background**. Self-congruency refers to the coherence between emotional experience (internal states) and enacted behavior (outward actions). Reduced self-congruency has been linked to vulnerability in mental health, yet its physiological correlates remain poorly characterized. Heart–brain temporal coupling may provide a candidate physiological marker of this psychological coherence. **Methods**. Thirty-eight healthy adults underwent resting-state functional magnetic resonance imaging while cardiac activity was simultaneously recorded using photoplethysmography to derive heart rate variability (HRV). Self-congruency was assessed using a graphic rating scale based on the spatial overlap between emotional experience and enacted behavior. Heart–brain temporal coupling between HRV and regional blood-oxygen-level-dependent (BOLD) signals was quantified using cross-covariance analysis across biologically plausible temporal shifts. **Results**. Heart–brain temporal coupling predominantly reflected brain-to-heart temporal ordering, particularly within regions central to the neurovisceral integration model, including the ventromedial prefrontal and anterior cingulate cortices. In contrast, higher self-congruency was associated with stronger heart-to-brain temporal coupling, notably within the right rostral middle frontal gyrus and supramarginal gyrus, regions implicated in emotion regulation and socio-emotional processing. **Conclusions**. While global heart–brain temporal coupling is dominated by top-down neural regulation, greater alignment between emotional experience and enacted behavior is associated with enhanced bottom-up cardiac temporal ordering on neural activity. These findings seem to identify a physiological–psychological axis that may inform original prevention-oriented approaches in mental health.

## 1. Introduction

The commonsense advice to “*follow your heart*” may be more than a metaphor. People often report greater well-being when their actions align with their inner emotions and values [1,2]. This alignment, referred to as self-congruency [3], captures the coherence between what individuals feel and how they act. Here, we conceptualized self-congruency as an individual difference that can be expressed as a relatively stable tendency, while acknowledging that it may also fluctuate with context and current affective state. High self-congruency is associated with positive life evaluations [3] and subjective well-being [4], underscoring its relevance for mental health as emphasized by the World Health Organization [5]. By contrast, reduced coherence between inner states and outward behavior is associated with emotional distress and regulatory difficulties [2,6]. Identifying physiological correlates of this coherence could therefore help to bridge subjective experience with objective markers relevant to mental health vulnerability and resilience.

The heart and the brain maintain continuous bidirectional communication, primarily through the vagus nerve, the major parasympathetic pathway. The neurovisceral integration model [7,8] proposes that the neural systems involved in emotion, cognition, and self-regulation exert top-down temporal ordering on the cardiovascular and respiratory physiology [9], while afferent cardiac signals reciprocally modulate brain states. Some evidence is available in adolescents [10], in patients with refractory epilepsy [11], and regarding fear-conditioning [12]. Consequently, fluctuations in thoughts and emotions shape cardiac dynamics while cardiac patterns influence perceptual, emotional, and behavioral responses [13]. Yet the temporal directionality of this interplay remains unclear. Furthermore, it is unknown whether individuals with higher self-congruency exhibit a distinctive balance within this heart–brain communication compared to individuals with lower self-congruency. Critically, distinguishing brain-leading (efferent regulatory or top-down) from periphery-leading (afferent/interoceptive or bottom-up) temporal ordering is central to current models of emotion regulation or interoception and could clarify which component of the heart–brain loop is most relevant to self-congruency. Establishing such temporal directionality may contribute to identifying physiological markers of psychological coherence associated with vulnerability in mental health.

Heart rate variability (HRV), the fluctuation in time intervals between successive heartbeats, reflects the dynamic interplay of sympathetic and parasympathetic influences on the heart. Since its formalization in the 1996 international consensus guidelines [14], HRV has been widely used as an index of autonomic regulation relevant to stress, cardiovascular health, and mental well-being [7,8,15]. Greater HRV indicates higher regulatory flexibility (i.e., the capacity of autonomic control to adjust efficiently to changing internal and external demands) [16]. This flexibility supports emotional stability, not as rigidity but as the capacity to maintain context-appropriate emotional states across fluctuations in environment and internal experience [17]. Individuals with higher resting HRV typically show stronger emotion regulation capacity and better mental health outcomes [6]. Although HRV is derived from beat-to-beat cardiac intervals, it primarily indexes autonomic modulations acting on the heart rather than intrinsic myocardial activity. Accordingly, HRV can be viewed as a regulated peripheral output summarizing central autonomic processing and reflexive regulation (e.g., baroreflex and respiratory sinus arrythmia), rather than as a purely bottom-up cardiac driver. HRV may therefore contribute to providing a physiological window into self-congruency, particularly because the construct reflects the degree of coherence between one’s emotional experience and enacted behavior. In this framework, self-congruency is expected to relate to integrative autonomic regulation (i.e., combined sympathetic-parasympathetic coordination) rather than to vagal tone alone.

Neural dynamics also show intrinsic temporal variability. Metastability (i.e., the fluctuation of phase synchronization among large-scale neural networks) captures the brain’s capacity to reconfigure its coordination patterns over time [18]. Such variability supports flexible information processing and underlies emotion regulation and cognitive control [19]. Evidence links higher HRV with greater cognitive flexibility [20], suggesting coordination between flexible cardiac and flexible neural systems. However, HRV and metastability are not expected to map one to one: HRV indexes peripheral autonomic regulation, whereas metastability captures distributed neural coordination that may be shaped by multiple non-autonomic factors (e.g., arousal, attention, and large-scale network organization). In line with the neurovisceral integration framework, low HRV is often associated with reduced adaptability [8,21]. Collectively, these findings support our working hypothesis: flexible autonomic and neural dynamics facilitate the integration of emotions and actions, a process that may underlie self-congruency and could be leveraged as a potential biomarker of psychological coherence and vulnerability in mental health.

**Figure 1:**
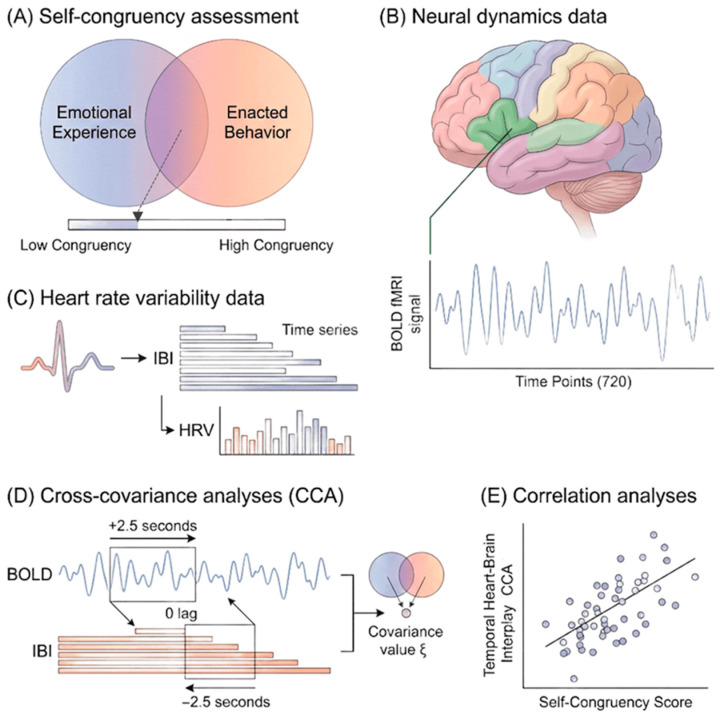
Schematic illustration of the methods used in this study. fMRI, functional magnetic resonance imaging; HRV, heart rate variability; IBI, inter-beat interval; BOLD, blood-oxygen-level-dependent; CCA, cross-covariance analysis. (**A**) Self-congruency assessment. Participants estimated their self-congruency using a graphic rating scale. Two cardboards representing their emotional experience and enacted behavior were overlapped, and the degree of overlap was quantified using a proportional metric (see Supplementary Figure S1). (**B**) Neural dynamics data. Resting-state fMRI was acquired with a repetition time (TR) of 500 ms, corresponding to a sampling frequency of 2 Hz and yielding 720 time points over 6 min. T1-weighted structural images were parcellated into 506 regions of interest based on the Lausanne 2018 symmetric atlas [29]. For each region, the mean BOLD fMRI signal was extracted across 720 time points. (**C**) Heart rate variability data. Photoplethysmography (PPG) was recorded at 200 Hz throughout the fMRI acquisition. Peak detection and interpolation yielded an IBI time series, from which HRV measures were derived. For analyses requiring temporal alignment with the BOLD signal, the IBI series was resampled at 2 Hz to match the fMRI sampling frequency. (**D**) Cross-covariance analyses (CCA). Temporal coupling between IBI and BOLD signals was quantified using CCA across temporal shifts ranging from −2.5 to +2.5 s. Negative shifts indicate BOLD preceding IBI; positive shifts indicate IBI preceding BOLD. Each CCA produced one covariance value (ξ). For each participant, 506 covariance values were obtained per shift. Eleven shifts were analyzed in total: five shifts with BOLD preceding IBI by 0.5–2.5 s (example shown for +2.5 s), five shifts with IBI preceding BOLD by 0.5–2.5 s, and one zero-lag condition (see Supplementary Figure S2). Because a 0.5-s shift corresponds to one fMRI time point, the overlap between signals varied by shift, resulting in CCA lengths ranging from 720 to 715 time points. (**E**) Correlation analyses. Associations between temporal heart–brain covariance and self-congruency were assessed by correlating (Bravais–Pearson coefficients) CCA values with participants’ self-congruency scores at each temporal shift.

Given that both self-congruency [3] and heart–brain dynamics relate to emotion (e.g., anxiety [15,21]), cognition, behavior, and well-being (i.e., self-regulation [4,6,8,13,22,23]), we asked whether individual differences in self-congruency track not only the magnitude but also the temporal ordering of heart–brain coupling. The present study therefore examined whether self-congruency is associated with the temporal coupling and directional flow of heart–brain interactions (see Figure 1). Because brain-leading effects are typically interpreted as top-down autonomic regulation, whereas periphery-leading effects can reflect interoceptive and reflex-related influences expressed at the heart (i.e., bottom-up temporal ordering), we explicitly tested both directions across biologically plausible delays [24]. In framing these analyses, we further considered whether such temporal signatures might reflect candidate physiological markers relevant for mental health. Based on previous work [8,13,25,26,27,28], we predicted three outcomes: (1) higher self-congruency would be positively related to greater HRV metrics, but not necessarily to neural metastability; (2) heart–brain covariance would involve regions central to the neurovisceral integration model; (3) greater self-congruency would be associated with stronger heart–brain temporal ordering (i.e., an heart brain temporal covariance), consistent with greater integration of autonomic/interoceptive signals within brain systems supporting self-regulation.

## 2. Materials and Methods

### 2.1. Procedure

The study was conducted in accordance with the Declaration of Helsinki and approved by the local Ethics Committee (Vaud CER PB_2016-02008, 204/15). Data were collected during a single 90-min session, ensuring that behavioral, physiological, and neuroimaging data reflected the same baseline state. Participants first received an oral explanation of the study procedures and provided written informed consent. The session included a behavioral and cognitive assessment followed by MRI acquisition of anatomical and resting-state functional data. A photoplethysmography (PPG) sensor (Siemens Prisma, Siemens, Erlangen, Germany) was placed on the tip of the right index finger, and pulse data were recorded simultaneously with the MRI acquisition.

### 2.2. Study Design

The present study relies on a within-individual physiological design, in which cardiac and neural signals are continuously measured and temporally coupled within each participant. Each participant therefore served as their own physiological reference, allowing the assessment of intrinsic heart–brain temporal organization. Rather than contrasting experimental conditions, the objective was to characterize individual variability in intrinsic heart–brain temporal organization during the resting state, a context known to reveal stable trait-like physiological signatures. Resting-state recordings were selected to probe baseline regulatory architecture rather than task-evoked responses, which may confound dispositional traits with transient cognitive or emotional changes (or states). Future work may extend this approach by examining task modulation; however, this was beyond the scope of the present investigation.

### 2.3. Participants

In total, 44 adults (29 females) were initially recruited for this study through word of mouth. Inclusion criteria were: age between 17 and 65 years, normal or corrected-to-normal vision, and absence of self-reported neurological, psychiatric, or cardiovascular disorders. Exclusion criteria included a history of neurological disease, current psychotropic medication, contraindications to MRI, or poor-quality physiological recordings. All participants were healthy volunteers with no history of neurological disorders. Data from 6 individuals were excluded due to technical issues with physiological recordings (*n* = 5) or data-processing failure (*n* = 1), resulting in a final sample of 38 adults (26 females; age range: 17.0–63.2 years; mean ± SD: 31.2 ± 12.5). All acquisitions took place in the afternoon to minimize circadian variability in autonomic function (mean time ± SD: 3.8 pm ± 2.1). Participants were tested at least two hours post-prandial, and body mass index (BMI) was within the normative range for the sample (mean ± SD: 21.22 ± 2.19, min–max: 17.3–26.2), and all were healthy and physically active, but not competing at regional level or above, reducing potential metabolic confounding on HRV measures.

### 2.4. Cognitive and Emotional Variables

Because cognitive abilities can influence HRV regulation [20], fluid intelligence was assessed using the Standard Progressive Matrices, a nonverbal reasoning test [29]. The task comprises five sets of twelve incomplete visual matrices to be completed within 15 min. For each item, participants select the correct missing element from several response options, with difficulty increasing across sets. Scores correspond to the total number of correct responses (maximum = 60), with higher scores indicating greater fluid intelligence.

Given that anxiety can also modulate HRV metrics (e.g., [15]), participants completed the short form of the State–Trait Anxiety Inventory (STAI-SF), developed by Marteau and Bekker (1992; [30]) based on Spielberger’s original instrument. The STAI-SF includes six items rated on a 1–6 scale, yielding total scores between 6 and 36. Higher scores reflect higher levels of self-reported anxiety. These measures were included to assess and control for individual differences that could potentially influence autonomic regulation independently of self-congruency.

### 2.5. Data Acquisition and Preprocessing

#### 2.5.1. Neural Data

MRI data were acquired on a Siemens 3T Magnetom Prisma scanner (Siemens, Munich, Germany) equipped with a 64-channel head coil at the BioMedical Imaging Center (CIBM), University Hospital of Lausanne (CHUV-UNIL, Switzerland). Participants wore ear protection to reduce scanner noise, and foam padding was used to minimize head motion. A high-resolution 3D T1-weighted anatomical image (MPRAGE; TR = 500 ms, TE = 2.47 ms; 208 slices; voxel size = 1 mm × 1 mm × 1 mm; flip angle = 8°) was acquired as the anatomical reference for a 6-min resting-state fMRI scan. Functional images were collected using a multiband gradient-echo echo-planar imaging sequence sensitive to blood-oxygen-level-dependent (BOLD) contrast (voxel size = 2.2 mm × 2.2 mm × 3 mm; multiband factor = 4; TR (repetition time) = 500 ms (2 Hz); 720 volumes; total duration = 6 min). The high temporal resolution (2 Hz) was chosen to allow fine-grained temporal alignment with cardiac signals.

Preprocessing was performed with the fMRIPrep automated pipeline [31], part of the NiPreps suite (ver. 20.2.8, NiPreps Documentation Team, 2020). Anatomical preprocessing included construction of a T1w reference image (conformed to RAS orientation and uniform voxel size), skull stripping, brain tissue segmentation, and nonlinear normalization to a standard template. Functional preprocessing involved generating a reference BOLD image, estimating head motion, and applying susceptibility distortion correction to account for field inhomogeneities. This standardized pipeline minimizes preprocessing-induced variability and enhances reproducibility.

From the preprocessed BOLD data, mean time series were extracted for each of the 506 anatomical regions of interest defined by the Lausanne 2018 symmetric atlas [32]. For each region, activity values were averaged across all 720 time points [33] to reconstruct regional neural dynamics (Figure 1B).

Neural metastability, a measure of neural dynamics variability and adaptive flexibility [18,34], was computed using an in-house MATLAB^®^ (R2019a, MathWorks, Natick, MA, USA) script. Metastability was defined as the standard deviation of the Kuramoto order parameter over time [35], yielding an individual metastability score for each participant. This measure captures large-scale coordination dynamics rather than local neural variability.

#### 2.5.2. Heart Rate Variability (HRV) Data

Photoplethysmography (PPG) was recorded at 200 Hz throughout the 6-min resting-state fMRI acquisition. Beat-to-beat intervals were extracted directly from the PPG signal. Because sampling rates below 250 Hz can introduce temporal jitter in peak estimation, the theoretical temporal precision at 200 Hz is limited to a 5 ms resolution. To refine peak localization, a second-order polynomial was fitted around each detected peak using four neighboring samples (two preceding and two following), and the heartbeat was defined as the maximum of the interpolated polynomial. A second-order interpolation was selected because it provides sufficient precision for peak localization without introducing the sensitivity to noise that can accompany higher-order fits. This approach improves temporal precision while preserving physiologically meaningful variability. We used inter-peak intervals as a surrogate for inter-beat intervals (IBIs), which were computed as the time difference between successive peaks (Figure 1C) and served as the basis for HRV analysis.

IBIs were processed [14] to identify and remove ectopic beats using both an automated detection algorithm and visual inspection [36]. The automatic algorithm detects potential ectopic beats based on deviation from a moving median and corrects them using a shape-preserving cubic interpolation to obtain normal-to-normal (NN) intervals. From these NN intervals, we extracted three standard HRV metrics: RMSSD, and spectral power in the low-frequency (LF; 0.04–0.15 Hz) and high-frequency (HF; 0.15–0.40 Hz) bands, expressed in ms^2^ [37,38]. RMSSD is a widely used time-domain index of vagally mediated HRV [39,40]. LF reflects a combination of sympathetic and parasympathetic influences, including baroreflex-driven activity, whereas HF primarily represents parasympathetic activity and respiratory sinus arrhythmia [39,40]. High-frequency (HF) power was interpreted as reflecting predominantly parasympathetic modulation rather than a direct measure of respiratory sinus arrhythmia, in line with recent recommendations emphasizing the influence of uncontrolled respiration on HF-HRV estimates. Notice that respiration was not recorded and may affect HRV-related measures.

Spectral power density was computed using a fast Fourier transform applied to NN intervals resampled at 2 Hz with a window length of 250 data points and 50% overlap. The 2 Hz resampling frequency matches the 720-time-point structure of the 6-min fMRI acquisition and adheres to recommended HRV analysis standards [14]. All HRV computations were performed in MATLAB^®^ (R2019a, MathWorks, Natick, MA, USA). The raw PPG waveform also contains information about peripheral vascular dynamics and breathing-related oscillations. These components were not analyzed in the present study, which was specifically focused on autonomic modulation indexed by HRV.

#### 2.5.3. Self-Congruency Data

Self-congruency was assessed using a graphic rating scale in which participants adjusted the overlap between two circular cardboards representing their *emotional experience* and *enacted behavior*. The graphic overlap method builds directly on a well-established tradition of visuospatial self-assessment tools, most notably the Inclusion of Other in the Self (IOS) scale [41], which has demonstrated strong validity across psychological and neurophysiological studies [42,43]. Participants were instructed to position the circles to reflect the extent to which they perceived these two aspects of themselves to match, explicitly instructed to report their *general and typical experience*, rather than a transient emotional state, thereby conceptualizing self-congruency as a relatively stable, trait-like characteristic. Greater overlap indicated higher self-congruency.

Both circles were identical in size, each with radius *r*. To quantify the overlap, the distance *d* between the centers of the two circles was measured (Figure 1A). The area of intersection was then computed using the standard formula:

$$A=r^{2}(\theta -sin\theta ),\theta =2arccos(\frac{2r-d}{2r})$$

(as detailed in Supplementary Figure S1). Because the aim was to express self-congruency as a simple percentage, this intersection area was converted into a proportional score by dividing it by the total area of one circle (*πr*^2^) and multiplying by 100, such that self-congruency directly reflected the proportion of overlap between emotional experience and enacted behavior.

### 2.6. Data Analysis

#### 2.6.1. Demographic, Group Variables and HRV Signal

Descriptive statistics for demographic variables and group measures were computed using RStudio (version 4.2.2). To assess whether anxiety or fluid intelligence was associated with inter-beat interval (IBI) time series, correlations were calculated at each of the 720 time points using MATLAB^®^ (R2022b, MathWorks, Natick, MA, USA). A significance threshold of *p* < 0.001 was applied, and multiple comparisons were corrected using the false discovery rate (FDR) procedure at 0.001 (Groppe, 2022, MATLAB Central File Exchange).

#### 2.6.2. Global Relations of Self-Congruency with HRV Metrics and Neural Metastability

For each participant, self-congruency, HRV metrics, and neural metastability were represented by global (time-averaged) values. Associations between self-congruency and each variable of interest (i.e., metastability, RMSSD, LF, and HF) were examined using correlation analyses across participants. Pearson or Spearman correlations were selected based on data normality. Analyses were conducted in RStudio (version 4.2.2), with *p* < 0.05 considered statistically significant. Correlations between HRV metrics and self-congruency were treated as targeted analyses grounded in strong a priori hypotheses; therefore, *p* < 0.05 (uncorrected) was used to avoid inflating Type II error. In contrast, cross-covariance analysis (CCA)-based temporal coupling analyses involved thousands of values across regions and shifts and were therefore subjected to FDR correction.

#### 2.6.3. Temporal Relation Between Neural Dynamics and HRV

Temporal coupling between neural dynamics and HRV was examined using CCA applied to the preprocessed BOLD time series and IBI signal resampled at 2 Hz. CCA estimates the similarity between two signals across a range of temporal delays. For each participant, CCA was computed across delays from −2.5 to +2.5 s (Figure 1D; Supplementary Figure S2), reflecting biologically plausible conduction delays between cardiac and neural activity [44]. The selected temporal window does not aim to capture the peak of the hemodynamic response to discrete neural events, but rather the relative timing of spontaneous BOLD fluctuations and cardiac intervals. Because resting-state BOLD signals already reflect hemodynamically filtered neural activity, additional Hemodynamic Response Signal (HRF) shifting or deconvolution was not applied. The ±2.5 s window was chosen to capture physiologically plausible delays [44,45] related to autonomic signaling and baroreflex-mediated feedback rather than stimulus-locked neural responses. Negative delays indicate the BOLD signal shifted ahead of the IBI signal, whereas positive delays indicate the IBI signal shifted ahead of the BOLD signal.

For each delay, 506 cross-covariance values were obtained, one for each brain region of interest, yielding eleven total delays: five with BOLD leading, five with IBI leading, and one zero-lag condition. For each region and delay, the reported value corresponds to the peak (maximum absolute) of the cross-covariance function within the overlapping segment of the two signals. To assess group-level effects, Wilcoxon signed-rank tests were performed at each delay across participants. Significance was defined as *p* (FDR-corrected) < 0.001 using the Benjamini–Hochberg procedure (fdr_bh). All CCA and nonparametric analyses were conducted in MATLAB^®^ (R2022b).

#### 2.6.4. Relation Between Heart–Brain Covariance and Self-Congruency

To explore whether heart–brain temporal coupling covariance was associated with self-congruency, correlation analyses were computed across participants using CCA magnitude as the dependent measure and self-congruency score as the predictor (Figure 1E). For each brain region and each predefined temporal shift, the peak value of the cross-covariance function within the overlapping segment of the IBI and BOLD signals was extracted. The signed covariance value was retained, preserving the direction of co-fluctuation between signals. Directionality of interaction (heart-to-brain vs. brain-to-heart in heart–brain temporal coupling) was determined by the sign of the temporal shift, not by transforming covariance values. Correlations were computed separately for each region and temporal shift. Brain regions showing associations at *p* < 0.001 were considered significant. Given that the correlations with self-congruency served as an exploratory brain–behavior analysis rather than a confirmatory test, we report results at an uncorrected threshold of *p* < 0.001 to avoid excessive Type II error while still applying a stringent criterion. These analyses were performed in MATLAB^®^ (R2022b).

#### 2.6.5. Correction for Multiple Comparisons—*p*-Value Adjustment

Different statistical correction strategies were applied depending on the scope and hypothesis structure of each analysis. Group-level CCA involved a large number of comparisons across brain regions (*n* = 506) and temporal delays (*n* = 11); therefore, FDR correction was applied across regions separately for each delay using the Benjamini–Hochberg procedure. By contrast, correlations between global HRV metrics and self-congruency were treated as targeted analyses grounded in strong a priori hypotheses derived from the neurovisceral integration framework. To avoid inflating Type II error, these correlations were therefore reported using an uncorrected significance threshold of *p* < 0.05. Exploratory brain–behavior correlations between regional CCA values and self-congruency were reported at a stringent uncorrected threshold of *p* < 0.001, consistent with their hypothesis-generating nature.

## 3. Results

### 3.1. Cognitive Variables, Emotional Variables, and HRV Signal

The final sample included thirty-eight participants whose demographic and behavioral characteristics are presented in Table 1. Rather than implying strict homogeneity, the absence of extreme values in fluid intelligence and anxiety scores indicates that the sample did not include individuals with atypical cognitive or affective profiles.

Across all 720 time points, inter-beat interval (IBI) values showed no significant association with anxiety levels (all *p* > 0.17, FDR-corrected) or fluid intelligence scores (all *p* > 0.20, FDR-corrected). These findings indicated that neither anxiety nor cognitive ability had a measurable temporal ordering on the HRV metrics used in this study.

Self-congruency scores showed substantial interindividual variability, ranging from 13.4% to 97.5%.

Potential effects of age and sex were explicitly examined: age was not correlated with self-congruency or HRV indices (RMSSD, LF, HF; all *p* > 0.54), nor did a multivariate analysis of covariance reveal significant effects of age or sex on self-congruency or HRV metrics (all multivariate *p* > 0.26), indicating that demographic factors did not account for the observed associations.

### 3.2. Global Relations of Self-Congruency with HRV Metrics and Neural Metastability

**Figure 2:**
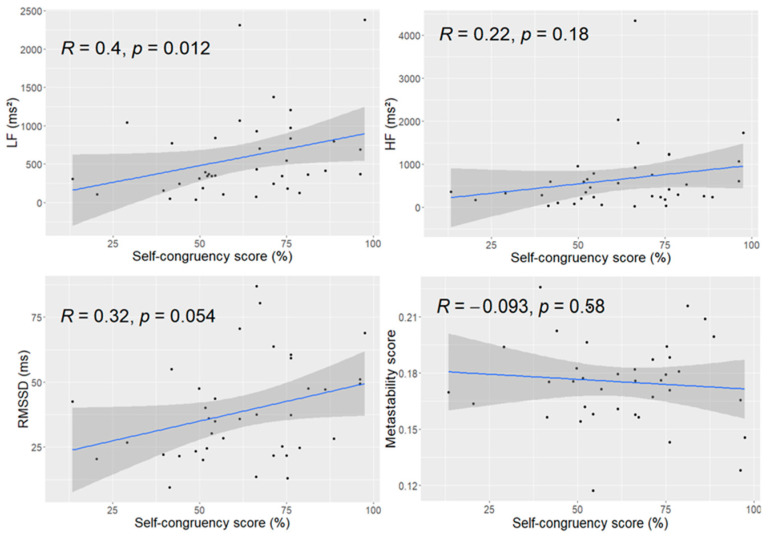
Associations between self-congruency, HRV metrics, and neural metastability. Self-congruency scores showed a significant positive correlation with low-frequency (LF) HRV measures (**top left panel**) and a trend-level association with RMSSD (root mean square of successive differences; **bottom left panel**). No significant associations were observed between self-congruency and high-frequency (HF) HRV measures (**top right panel**) or neural metastability values (**bottom right panel**). LF, low frequency; RMSSD, root mean square of successive differences; HF, high frequency.

Linear correlations between measures of HRV and neural metastability with self-congruency scores are shown in Figure 2. Only the LF band measure was positively correlated with the self-congruency score (*r* (36) = 0.40, *p* = 0.01). Despite a positive trend, the RMSSD was not significantly correlated with the self-congruency score (*r* (36) = 0.32, *p* = 0.054). No significant linear correlation was also observed between the self-congruency score and the HF band measure (*r* (36) = 0.22, *p* > 0.1), nor the metastability score (*r* (36) = −0.093, *p* > 0.5). These results suggest that self-congruency relates preferentially to integrative autonomic indices rather than to parasympathetic tone alone or to global neural coordination.

### 3.3. Temporal Relation Between Neural Dynamics and HRV

**Figure 3:**
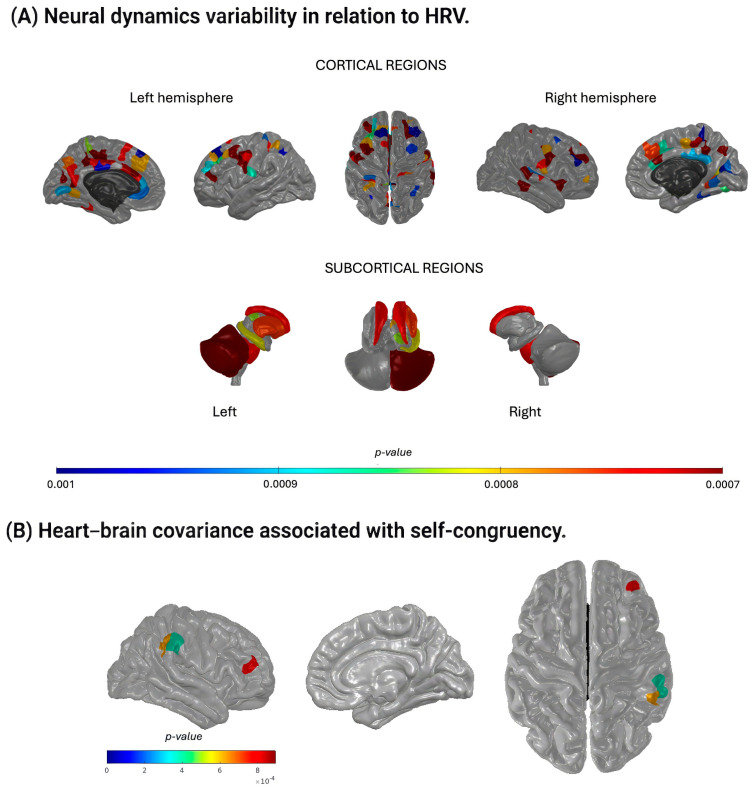
Neural-cardiac relations and their association with self-congruency. Abbreviations: HRV, heart rate variability; IBI, inter-beat interval; BOLD, blood-oxygen-level-dependent signal; FDR, false rate discovery. (**A**) Neural dynamics variability in relation to HRV. Brain regions in which inter-beat interval (IBI) fluctuations significantly covaried with BOLD activity when the BOLD signal was shifted 1 s prior to the IBI signal are shown for cortical (top) and subcortical (bottom) structures. Subcortical regions are displayed in detail in the lower panel. The color scale represents *p*-values, and regions surviving a threshold of *p* < 0.001 (FDR-corrected) are shown. (**B**) Heart–brain covariance associated with self-congruency. Brain regions in which IBI–neural activity covariance positively correlated with self-congruency scores are displayed. Covariance values correspond to the condition in which the IBI signal was shifted 2.5 s before the BOLD signal. The color scale represents *p*-values, with regions significant at *p* < 0.001 (uncorrected).

The temporal relation between neural dynamics variability and HRV revealed significant covariance when the two signals overlapped (t = 0) or when the BOLD signal preceded the IBI signal by 0.5–2.5 s, indicating predominant brain-to-heart directionality (88 regions at *p* < 0.001; see Supplementary Figure S3). This pattern was consistent across delays, suggesting a stable group-level temporal architecture rather than delay-specific effects. Notably, the set of regions showing significant covariance when the BOLD signal was shifted 1 s before the IBI signal largely overlapped with those identified at all other significant shifts (Figure 3A; Supplementary Figure S3), reflecting a robust and consistent temporal pattern.

Among the eighty-eight regions showing significant covariance at the 1-s shift (Supplementary Table S1), several key functional systems were represented. These included components of the basal ganglia (e.g., caudate, putamen), subcortical structures (e.g., thalamus, hippocampus), the insular cortex, and widespread cortical regions. Importantly, these regions largely overlap with nodes of the central autonomic network and default-mode adjacent regulatory systems rather than primary sensory or motor cortices, consistent with their role in autonomic integration and emotion regulation [46,47,48,49]. Specifically, thirty-three regions were located in the frontal lobe (e.g., rostral middle frontal, caudal middle frontal, superior frontal), nineteen in the parietal lobe (e.g., superior parietal, inferior parietal, precuneus), twelve in the limbic lobe (e.g., rostral and caudal anterior cingulate), eleven in the occipital lobe (e.g., cuneus, fusiform), and five in the temporal lobe (e.g., middle and superior temporal gyri). The correlations between self-congruency and heart–brain temporal covariance for these regions are further illustrated in Supplementary Figure S4.

### 3.4. Relation Between Heart–Brain Covariance and Self-Congruency

Three regions showed a significant positive correlation between heart–brain covariance and self-congruency when variability in IBIs, indexing autonomic modulation of cardiac activity, preceded regional BOLD fluctuations by 2.5 s, consistent with a heart-to-brain temporal ordering (Table 2). Here, covariance magnitude (not temporal lag) was correlated with self-congruency, while lag was fixed a priori to reflect physiologically plausible autonomic delays. This temporal window aligns with the typical latency of arterial baroreflex-mediated cardiovascular adjustments (approximately 2–3 s at resting heart rates), consistent with the observed stronger LF-BOLD associations relative to HF or RMSSD indices.

Higher self-congruency was associated with stronger heart-to-brain temporal coupling covariance in the right rostral middle frontal gyrus (*r* (36) = 0.52, *p* < 0.001; Figure 3B) and in two sites within the right supramarginal gyrus (*r* (36) > 0.51, *p* < 0.001; Figure 3B). These associations were continuous across individuals rather than indicating discrete subgroups, suggesting graded variability in the integration of autonomic feedback within socio-emotional regulatory regions. These findings indicate that individuals whose emotional experience and enacted behavior are more aligned show enhanced cardiac temporal ordering on neural activity within regions implicated in emotion regulation and socio-emotional processing.

## 4. Discussion

The present study examined whether temporal signatures of heart–brain temporal coupling relate to self-congruency, defined as the coherence between one’s emotional experience and enacted behavior. Although prior research has explored bidirectional heart–brain temporal coupling communication [13,25], no previous work has characterized the temporal covariance between their respective signals nor tested whether this dynamic links to self-congruency. Here, we extend existing heart–brain frameworks by explicitly focusing on the timing and directionality of spontaneous heart–brain temporal coupling and by relating this temporal organization to an individual-difference construct relevant to mental health, namely self-congruency. Unlike self-awareness, which refers to the capacity to perceive or monitor one’s internal states, self-congruency concerns the perceived alignment between those internal emotional states and one’s enacted behavior. It also differs from authenticity or self-alignment, which are often framed in moral, identity-based, or value-driven terms and emphasize consistency with abstract self-ideals rather than moment-to-moment emotional–behavioral coherence, and not equivalent to self-concept clarity, which indexes the stability or consistency of self-beliefs across contexts. By contrast, the present construct specifically targets the experiential fit between felt emotion and outward action, positioning self-congruency as a functional, integrative marker of emotional regulation rather than a broader evaluative or identity-based trait.

Our findings indicate that although group-level patterns reflect predominant top-down brain-to-heart temporal ordering, the higher the self-congruency the stronger the heart-to-brain temporal coupling. Rather than identifying discrete subgroups, this pattern suggests the existence of a continuum of temporal heart–brain activity profiles, with enhanced bottom-up sensitivity emerging at the higher end of self-congruency. Importantly, this continuum-based interpretation avoids categorical classification and is consistent with dimensional models of psychological functioning. While preliminary, such variability hints at physiological signatures that could, in future work, inform the identification of individuals whose neurophysiological alignment or misalignment may carry relevance for mental health vulnerability. Indeed, both physiological regulation and psychological coherence are central to mental health [3,4,8,13,15,21,22,50,51], and clarifying how these domains intersect may help to identify candidate dimensions for biomarker-informed prevention rather than diagnostic classification.

Self-congruency was positively associated with LF power, a marker influenced by both sympathetic and parasympathetic activity and tightly linked to baroreflex function [39,40]. Enhanced baroreflex engagement is often interpreted as reflecting good self-regulatory capacity [8]. Rather than indexing stress-related sympathetic activation, LF power in the present resting, supine context likely reflects integrative autonomic control mechanisms. This suggests that greater coherence between emotional experience and enacted behavior may accompany stronger integrative autonomic regulation. RMSSD showed a positive trend in the same direction, whereas HF power (i.e., an index of vagal tone) did not relate to self-congruency. The absence of HF effects is consistent with recent work questioning the specificity of HF as a parasympathetic marker in the absence of respiratory control. These findings support the view that self-congruency aligns more closely with integrated autonomic flexibility than with parasympathetic activity alone. However, future studies should control for respiration rate to have a more precise view of HF influence.

A few physiological factors should temper interpretation of the LF–self-congruency association. Baroreflex sensitivity declines with age, raising the possibility that age-related autonomic variation could influence LF power; although age did not correlate with self-congruency in this sample, future studies should explicitly model age-related baroreflex variation. In addition, recordings were acquired in the supine position, where baroreflex engagement is relatively reduced due to minimal hydrostatic load. Thus, the observed effects likely represent conservative estimates of heart–brain coupling under low postural demand. However, posture-dependent effects therefore cannot be ruled out and should be examined in follow-up work.

Neural metastability, a marker of dynamic coordination across large-scale networks [18,34], showed no association with self-congruency. This null finding contrasts with the exploratory hypothesis that more flexible neural coordination might parallel alignment between emotional experience and enacted behavior. This result suggests that self-congruency may not depend on global neural reconfiguration capacity per se, but rather on the integration of interoceptive signals within specific regulatory networks, and may depend more strongly on interoceptive or affective signaling than on global fluctuations in neural synchrony. Future studies should examine additional indices of neural variability, especially those directly tied to emotion-related circuitry, including network-specific metastability or interoceptive network dynamics.

A central aim of this study was to characterize the temporal relationship between cardiac and neural fluctuations. Group-level analyses showed that covariance emerged predominantly when neural activity coincided with or preceded cardiac fluctuations, reflecting a strong brain-to-heart temporal ordering. This finding aligns with well-established models of vagal regulation, in which prefrontal and cingulate regions orchestrate autonomic output through the central autonomic network [8,28]. This dominant top-down pattern likely reflects baseline autonomic control during resting state rather than task-evoked or reactive processes. Consistent with these models, significant covariance involved regions highly implicated in autonomic and emotion regulation, including the ventromedial prefrontal cortex, anterior cingulate cortex, thalamus, insula, and basal ganglia, mirroring patterns reported in prior work using alternative analytic approaches [25,50]. These convergent findings underline the robustness of a physiological architecture wherein fluctuations in cardiovascular activity are closely tethered to neural systems that support self-regulation.

Although this top-down temporal ordering characterized the group, an important interindividual distinction emerged. Higher self-congruency was associated with stronger heart–brain temporal covariance specifically when cardiac activity preceded neural fluctuations by 2–2.5 s. This latency corresponds to the typical timescale of baroreflex-mediated cardiovascular feedback rather than to hemodynamic response delays. Given that HRV reflects autonomic regulation of the heart rather than intrinsic pacemaker activity, these heart-preceding-brain effects are best interpreted as the timing of autonomic adjustments captured peripherally at the heart, rather than direct myocardial signals driving neural responses.

Three right-hemisphere regions drove this association: two subregions of the supramarginal gyrus and one of the rostral middle frontal gyrus. The supramarginal gyrus supports socio-emotional functions such as empathy and the ability to differentiate self from other perspectives [44,52]. The rostral middle frontal gyrus, within the dorsolateral prefrontal cortex, plays a central role in emotion regulation, cognitive control, and empathy-related processing [53,54,55,56]. The observed associations converge on nodes of the default mode and central autonomic networks, systems known to integrate interoceptive signals with self-referential and regulatory processes [49,57,58]. Importantly, the involvement of these regions is not arbitrary but reflects their privileged anatomical and functional position within cortico-brainstem autonomic pathways. Both the rostral middle frontal gyrus and the supramarginal gyrus are structurally and functionally connected, directly or indirectly, to brainstem nuclei implicated in autonomic and respiratory control, including the nucleus tractus solitarius, parabrachial complex, and periaqueductal gray. These brainstem structures relay visceral afferent information to higher-order cortical regions via thalamic and insular hubs, forming the backbone of the central autonomic network. In this framework, enhanced heart–brain temporal coupling may index increased sensitivity of self-relevant neural systems to bodily signals, consistent with contemporary models of interoceptive inference. The exclusive involvement of right-lateralized regions is consistent with theoretical accounts highlighting right-hemisphere specialization for affective, autonomic, and integrative processing [8,23,28].

The temporal ordering of these associations (i.e., heart-preceding-brain), suggests that individuals with greater coherence may integrate cardiac signals more robustly into neural systems governing emotional and social cognition. This interpretation is consistent with, though not demonstrating, directional temporal ordering. While causal inference cannot be drawn from covariance analyses, this pattern is consistent with enhanced autonomic–cortical integration rather than reverse directionality. Physiological cues from the heart may contribute to shaping neural processing in individuals whose actions and emotional experience are more coherent, potentially supporting self-regulation. This view dovetails with broader evidence linking emotional coherence, self-regulatory capacity, and mental health [13,59]. Given the global burden of mental health disorders [5], physiological signatures that reflect emotional–behavioral alignment may hold translational potential for assessing well-being or monitoring intervention effects, pending replication in larger or clinical cohorts.

Several exploratory considerations emerged. Preliminary observations suggested slightly higher self-congruency among men in this sample, though larger samples are required to evaluate gender differences in heart–brain temporal coupling. Developmental research would also be informative, as HRV and neural dynamics change markedly from childhood through adulthood [60]. Further, integrating gut physiology, a system often intuitively linked to decision-making, may advance understanding of broader interoceptive contributions to emotional coherence.

Limitations should be noted. The graphic self-congruency measure used here should be viewed as a parsimonious, continuous proxy rather than a fully standardized psychometric instrument. Although similar graphical overlap methods have been employed to capture perceived self-alignment and coherence [43], formal validation and test–retest reliability remain to be established. Future studies should examine convergent validity with established measures of authenticity, self-concept clarity, or value–behavior congruence. Additionally, while physiological signal quality was carefully controlled using automated detection and manual inspection, the number and proportion of corrected beats were not systematically archived and thus cannot be retrospectively reported; datasets with excessive noise were excluded, leading to the removal of five participants. We selected RMSSD, LF and HF among the many HRV indices to distinguish between sympathetic and parasympathetic modulations to the heart, and address whether the heart–brain associations would be mediated by the parasympathetic branch of the autonomic nervous system. Respiration, which influences HF-HRV and may shape heart–brain associations [25,61], was not recorded, which limits the interpretation of HF power, particularly given recent recommendations to explicitly account for respiratory frequency [62,63,64]. Although HF remains informative regarding parasympathetic modulation, future studies should incorporate respiratory monitoring to dissociate cardiac autonomic influences from respiratory-driven variability. Although PPG sampling introduces slightly lower precision in beat-to-beat timing than ECG, PPG contains additional information about peripheral vascular dynamics that could be leveraged in future analyses to dissociate central cardiac modulation from vascular influences. The present study focused on time-domain cross-covariance, which quantifies the strength and timing of shared fluctuations between neural and cardiac signals. Complementary spectral or time-frequency descriptions could characterize the frequency structure of these shared dynamics, although they would not replace covariance as a measure of association strength. Integrating these approaches in future work may offer a more complete picture of multiscale heart–brain coupling. Covariance-based analyses of BOLD and HRV-derived series do not allow causal inference of directionality. Also, because HRV indexes autonomic regulation rather than intrinsic cardiac output, references to “*cardiac influence*” in this work should be understood as reflecting autonomic feedback captured at the heart, rather than direct myocardial signals acting on the brain. Finally, major confounders were minimized; however, factors such as individual fitness level, socio-economic background, or hormonal status were not explicitly modeled and may contribute residual variability. Future studies should explicitly address these factors to extend and refine the present findings.

In summary, individuals who report stronger coherence between their emotional experience and enacted behavior exhibit enhanced cardiac temporal ordering on neural regions supporting emotion regulation and social–emotional processing. Although the group-level pattern reflects predominant top-down control, interindividual variation in bottom-up cardiac sensitivity appears to track psychological coherence. The familiar advice to “*follow your heart*” acquires empirical nuance here: coherence between the heart’s signals and the brain’s regulatory systems may be a meaningful feature of emotional well-being. To conclude, whereas the present findings do not establish a diagnostic biomarker, it identifies a candidate physiological marker that may contribute to future multi-marker approaches in mental health assessment.

## Figures and Tables

**Table 1:** Descriptive statistics of participants.

Gender F [n]; M [n]	Age [Years]	Fluid Intelligence [Score/60]	Anxiety Level [Score/36]	Self-Congruency [Score %]
26; 12	31.2 ± 12.5 (17.0–63.2)	49.5 ± 6.0 (37.0–60.0)	13.0 ± 5.5 (6.0–25.0)	62.2 ± 19.9 (13.4–97.5)

Note. Data are mean ± SD (min–max). F Females, M Males.

**Table 2:** Relation between heart–brain temporal coupling covariance and self-congruency.

Brain Regions	Shifts (s)			
	2		2.5	
	r	*p* (10^−4^)	r	*p*
rh-rostral middle frontal subregion 6	-	NS	0.52	<0.001
rh-supramarginal subregion 3	-	NS	0.52	<0.001
rh-supramarginal subregion 5	0.52	8.41	0.54	<0.001

Note. *p*-values are expressed in units of 10^−4^ (all uncorrected). rh, right hemisphere. NS, non-significant. Covariance values correspond to the signed peak of the cross-covariance function at each predefined temporal shift.

## Data Availability

Data will be shared by the authors upon request to solange.denervaud@epfl.ch.

## Supplementary Materials

The following supporting information can be downloaded at: https://www.mdpi.com/article/10.3390/biomedicines14030548/s1, Figure S1: Schematic illustration of self-congruency assessment; Figure S2: Schematic illustration of cross-covariance analyses; Figure S3: The global relation between the BOLD and the IBI signals at all shifts (x-axis); Figure S4: Correlation results between self-congruency and heart-brain covariance; Table S1: Table summarizing the eighty-eight brain regions correlated to the heart signal at the shift presented in Figure 3.
