# Supplementary material for: Heart–Brain Temporal Coupling as a Candidate Biomarker of Self-Congruency
Source: Biomedicines. 2026 Feb 27;14(3):548. doi: 10.3390/biomedicines14030548 (PMC13023749; doi:10.3390/biomedicines14030548)
Supplement: Supplementary file 1 [file biomedicines-14-00548-s001.zip › biomedicines-4070751-supplementary.pdf]

## SUPPLEMENTARY INFORMATION

# Heart–Brain Temporal Coupling as a Candidate Biomarker of Self-Congruency


Nicolas Bourdillon 1,2,†, Sébastien Urben 3,†, Nina Rimorini 4, Alicia Rey 4, Cyril Besson 1,5, Jean-Baptiste Ledoux 4, Yasser Alemán-Gómez 6, Eleonora Fornari 4 and Solange Denervaud 4,7,*

1 Institute of Sport Sciences, University of Lausanne (UNIL), 1015 Lausanne, Switzerland

2 Teaching and Research Unit in Physical Education and Sport (UER EPS), University of Teacher Education (HEP), 1007 Lausanne, Switzerland

3 Division of Child and Adolescent Psychiatry (SUPEA), Department of Psychiatry, Lausanne University Hospital (CHUV-UNIL), 1011 Lausanne, Switzerland

4 CIBM Center for Biomedical Imaging, Department of Radiology, Lausanne University Hospital (CHUV) and University of Lausanne (UNIL), 1015 Lausanne, Switzerland

5 Sports Medicine Center, Swiss Olympic Medical Center, Lausanne University Hospital (CHUV), 1011 Lausanne, Switzerland

6 Department of Diagnostic and Interventional Radiology, Lausanne University Hospital (CHUV) and University of Lausanne (UNIL), 1011 Lausanne, Switzerland

7 MRI imaging and technology, Polytechnical School of Lausanne, Swiss Federal Institute of Technology Lausanne (EPFL), 1015 Lausanne, Switzerland

* Correspondence: solange.denervaud@epfl.ch

† These authors contributed equally to this work.


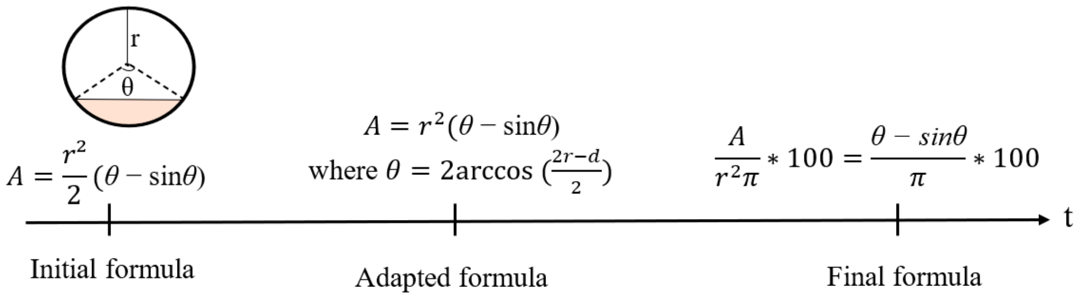


**Supplementary Figure S1** – Schematic illustration of self-congruency assessment.

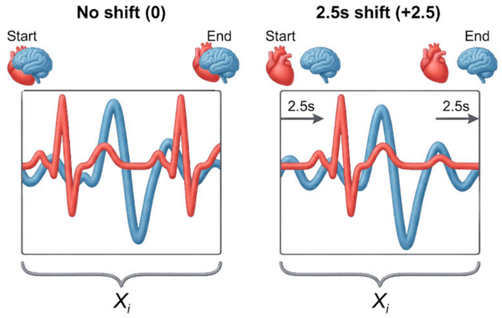


**Supplementary Figure S2 – Schematic illustration of cross-covariance analyses**. The shifts represented depict the analyses done when both signals were perfectly overlayed (illustrated on the left), and when the IBI signal was shifted from 0.5 to 2.5 seconds before the BOLD signal (illustrated for the 2.5 seconds shift; on the right).

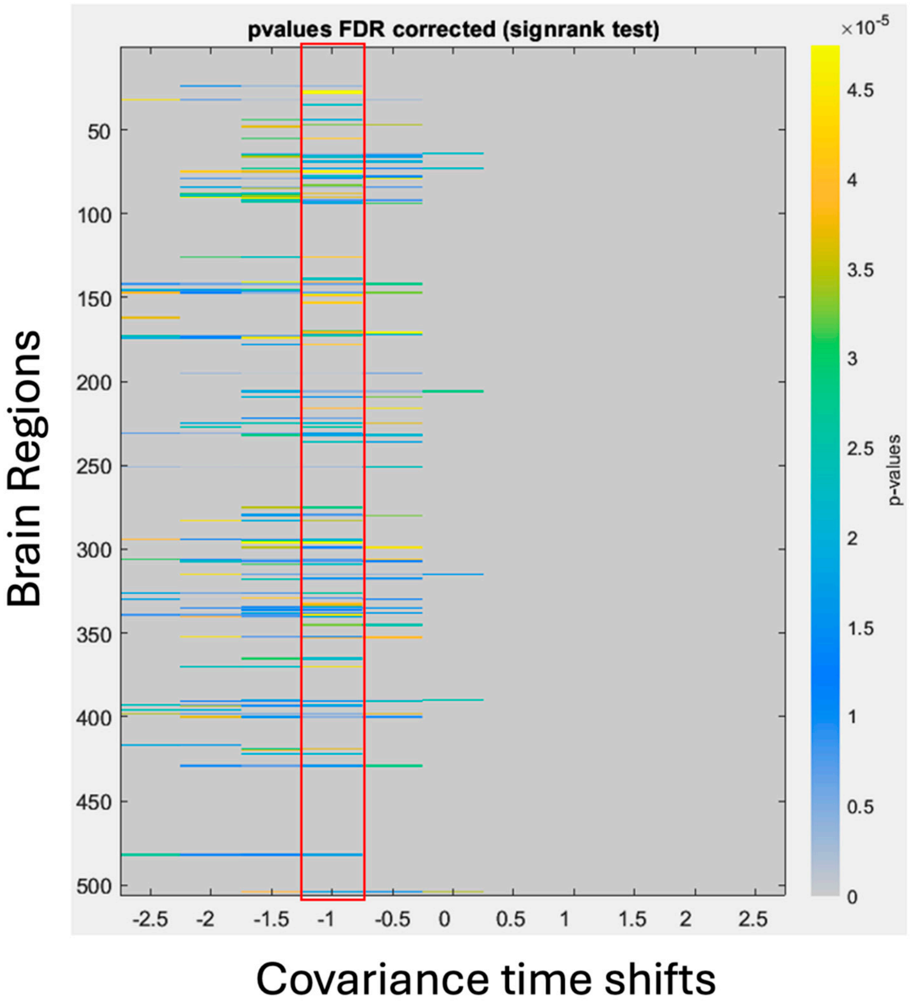


**Supplementary Figure S3 – The global relation between the BOLD and the IBI signals at all shifts (x-axis).** The red frame shows the results presented in Fig. 3. The color scale represents *p*-values. Brain regions (y-axis) are significant at $p<0.001$ FDR-corrected.

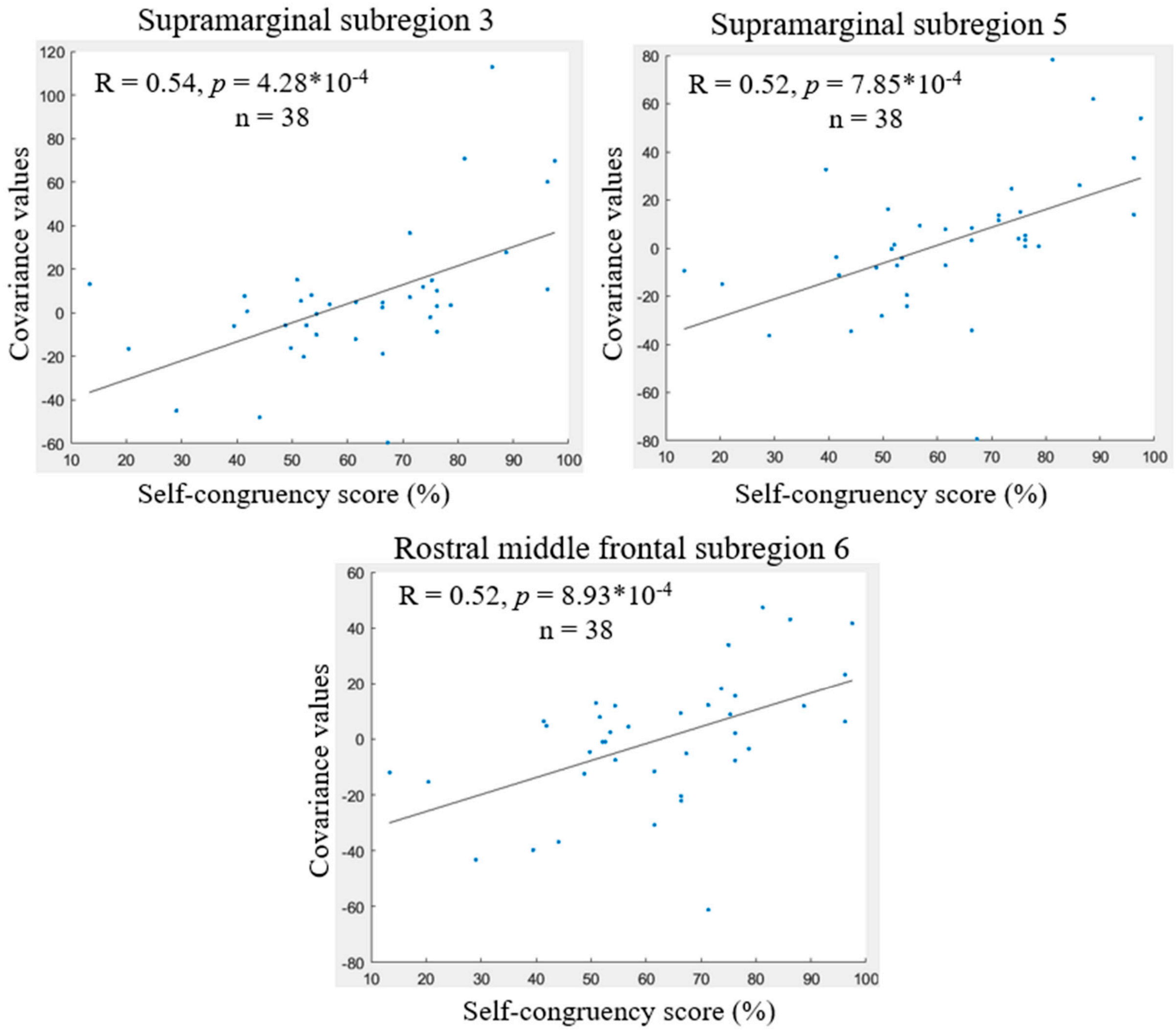


**Supplementary Figure S4 – Correlation results between self-congruency and heart-brain covariance**. Results for supramarginal subregion 3 (top left panel), supramarginal subregion 5 (top right panel), and rostral middle frontal subregion 6 (bottom panel).

**Supplementary Table S1 – Table summarizing the eighty-eight brain regions correlated to the heart signal at the shift presented in Fig. 3**, and framed in red in Supplementary Figure S3.

| | | Brain regions | | | |
|---|---|---|---|---|---|
| **rh** | **lh** | | **rh** | **lh** | |
| **n°** | **n°** | **Name** | **n°** | **n°** | **Name** |
| 24,27,28,32,35 | 275,279,280,283 | ctx-rostralmiddlefrontal | 153 | - | ctx-pericalcarine |
| 44,47 | 294,295,296,299 | ctx-superiorfrontal | 170,171,172 | 419,422 | ctx-lingual |
| 55 | 306,307,309 | ctx-caudalmiddlefrontal | 173,178 | 429 | ctx-fusiform |
| 64,65,66,69,73 | 315,317,318 | ctx-precentral | 195 | - | ctx-middletemporal |
| 75,78,79 | 326,329,330 | ctx-paracentral | 206,209 | - | ctx-superiortemporal |
| - | 332,333 | ctx-rostralanteriorcingulate | 216 | - | ctx-transversetemporal |
| 83,84 | 334,335,336 | ctx-caudalanteriorcingulate | 222 | - | ctx-insula |
| 88 | 338,339,340 | ctx-posteriorcingulate | 225 | - | thal-anterior |
| 90 | - | ctx-isthmuscingulate | 227 | - | thal-ventral_latero_dorsal |

| | | | | | |
|---|---|---|---|---|---|
| 92,93,94 | 345,352,353 | ctx-postcentral | 231 | 482 | subc-caudate |
| - | 365, 370 | ctx-superiorparietal | 232 | - | subc-putamen |
| 126 | - | ctx-inferiorparietal | 236 | - | subc-hippocampus |
| 139,141,142,147 | 390,391,393,394,398 | ctx-precuneus | 251 | - | cer-cerebellum |
| 148,149 | 400 | ctx-cuneus | | 504 | brain-stem-pons |
